# Research on the optimization of the training system of university faculty development centers in the context of GenAI: a comparative analysis based on Chinese and Kazakhstani universities

**DOI:** 10.3389/fpsyg.2026.1890508

**Published:** 2026-09-09

**Authors:** Tao Bi, Shakirova Araily, Anqi Lyu, Si Xiu

**Affiliations:** Al-Farabi Kazakh National University, Almaty, Kazakhstan

**Keywords:** comparison study, faculty development, GenAI pedagogical readiness, higher education, structured GenAI prompt-task training

## Abstract

**Introduction:**

This mixed-methods field study examined generative artificial intelligence (GenAI) pedagogical readiness among university faculty in five participating universities in China and Kazakhstan, with particular attention to cross-sample differences and the effects of faculty development training.

**Methods:**

Baseline survey data were collected from 568 faculty members, including 340 from China and 228 from Kazakhstan. Readiness was assessed across seven dimensions: basic GenAI understanding, prompt design, AI-supported instructional design, AI-supported assessment, critical judgement, educational ethics, and disciplinary transfer. A quasi-experimental subsample of 160 faculty members compared structured GenAI prompt-task training with conventional GenAI-focused faculty development. Prompt logs were additionally coded to identify post-training faculty development needs.

**Results:**

At baseline, the Kazakhstani sample reported higher readiness across all seven dimensions, with the largest differences in disciplinary transfer, prompt design, and basic understanding. Measurement-invariance analysis supported cross-sample comparison of observed mean patterns. In hierarchical regression, the Kazakhstan coefficient decreased from *B* = 0.205 to *B* = 0.041 after prior GenAI use, recent AI-related training, multilingual resource access, institutional support, perceived permission, policy clarity, risk sensitivity, and disciplinary background were included. Post-test comparisons showed greater readiness in the structured prompt-task training group than in the conventional group, particularly for prompt design and critical judgement. Prompt-design gains remained above baseline at delayed testing. Prompt evidence linked these gains to objective specification, constraint setting, assessment criteria, faculty verification, and risk-control prompts.

**Discussion:**

The findings indicate that GenAI pedagogical readiness is shaped by faculty course tasks, institutional support, resource access, and training design. Structured prompt-task training may therefore provide a useful approach to strengthening course-level faculty readiness for pedagogical uses of GenAI.

## 1 Introduction

Generative artificial intelligence (GenAI) has become part of routine higher education teaching. Faculty now encounter generated cases, classroom activities, reading materials, assessment rubrics, feedback texts, and student AI-use rules in course preparation. These materials are pedagogically useful when faculty can judge accuracy, disciplinary fit, learner appropriateness, privacy, academic integrity, and assessment fairness before using them (Kasneci et al., 2023; Lee et al., 2024; Wang et al., 2024). Faculty development centers therefore face a course-design problem: helping faculty turn generated outputs into verifiable, revisable, and accountable teaching practice.

This problem is especially visible in assessment. AI-assisted writing, coding, summarization, translation, and feedback weaken the evidentiary value of final products when assessment design remains unchanged. Assessment research emphasizes process evidence, authentic tasks, students' explanatory ability, and transparent rules for AI assistance (Foung et al., 2024; Xia et al., 2024; Luo, 2024). Multicultural studies further show that faculty and students interpret GenAI benefits, academic integrity, and institutional rules differently (Al-khresheh, 2024; Yusuf et al., 2024). GenAI pedagogical readiness is therefore a practical competence: faculty must formulate tasks, detect errors or bias, revise generated materials, redesign criteria, and explain permitted, disclosed, and prohibited uses.

China and Kazakhstan provide a comparative setting for examining how system-level digital education agendas reach course practice. China's smart-education agenda emphasizes national education digitalization, platform-based resources, AI-supported reform, and digital literacy (Ministry of Education of the People's Republic of China, 2025). Kazakhstan's AI agenda emphasizes infrastructure, human-capital development, university AI specializations, KazLLM development, and competence-building programs (Prime Minister of the Republic of Kazakhstan, 2024). Comparative education research treats national context as one explanatory layer for classroom practice (Bray et al., 2007). In the GenAI context, policy documents, platforms, and model ecosystems become relevant through local training provision, tool accessibility, multilingual resources, policy clarity, risk interpretation, and discipline-based support (Wang et al., 2024; Jin et al., 2025; An et al., 2025; McDonald et al., 2025; Sutedjo et al., 2025).

This study defines GenAI pedagogical readiness as university faculty members' capacity to use, evaluate, and regulate GenAI in course practice. The construct includes basic GenAI understanding, prompt design, AI-supported instructional design, AI-supported assessment, critical judgement, educational ethics, and disciplinary transfer. Drawing on data from five participating universities, the analysis combines a baseline comparison, a quasi-experimental training evaluation, and prompt-level qualitative analysis to examine how readiness is associated with exposure, training experience, resource access, institutional support, perceived permission, policy clarity, risk sensitivity, and disciplinary background, and how structured GenAI prompt-task training compares with conventional GenAI-focused faculty development.

## 2 Related work

### 2.1 Faculty development as situated professional learning

Faculty development matters when professional learning enters course design, classroom enactment, and assessment judgement. Research on professional development emphasizes sustained, active, content-focused learning connected to instructional work (Borko, 2004; Desimone, 2009). Guskey (2002) linked teacher learning, classroom implementation, and evidence of student outcomes within a single change process, and higher education development work helps faculty translate institutional quality agendas into course practice (Amundsen and Wilson, 2012).

GenAI extends this situated view because faculty development centers now support decisions about how generated content is requested, checked, revised, assessed, disclosed, and governed. Studies of faculty perceptions show that instructional benefit, academic integrity, privacy, classroom fit, anxiety, and professional development needs are intertwined (Cabellos et al., 2024; Almisad and Aleidan, 2025; Verano-Tacoronte et al., 2025). Reviews of higher education AI research also show weaker attention to educators' practical knowledge than to AI as a teaching technology (Zawacki-Richter et al., 2019; Sperling et al., 2024), while faculty profiles differ in technical confidence, perceived usefulness, and development needs (Mah and Groß, 2024). This literature positions faculty development as repeated practice with authentic GenAI-supported course tasks (Han, 2025; Prilop et al., 2025).

### 2.2 From AI literacy to GenAI pedagogical readiness

AI literacy provides a base for studying faculty competence: understanding AI concepts, using systems, evaluating outputs, recognizing risks, and making responsible decisions (Long and Magerko, 2020; Ng et al., 2021). Educator competence frameworks add a professional structure. TPACK links technology use with pedagogical and content knowledge (Mishra and Koehler, 2006); DigCompEdu connects digital competence with teaching, assessment, learner support, and responsible use (Redecker, 2017); UNESCO's AI competency framework places human-centered judgement, pedagogy, ethics, and professional learning in the teacher competence agenda (Miao and Holmes, 2024).

Educator-facing AI literacy research has moved from general awareness toward teaching practice. Scoping reviews highlight conceptual knowledge, technical use, and ethical awareness, while practical knowledge, classroom judgement, and disciplinary adaptation receive less attention (Sperling et al., 2024). Empirical studies link AI literacy and adoption to professional development, community support, perceived usefulness, technical experience, confidence, and anxiety (Ayyoub et al., 2025; Mah and Groß, 2024; Granström and Oppi, 2025). Intelligent-TPACK research similarly emphasizes connections among technical capability, pedagogical reasoning, and disciplinary purpose (Celik, 2023). GenAI pedagogical readiness builds on this work by focusing on whether faculty can judge, revise, assess, and regulate generated materials in course practice.

### 2.3 GenAI in higher education: instructional support, assessment pressure, and responsibility

GenAI research in higher education has moved from tool affordances to institutional responsibility. Faculty and students use GenAI to generate resources, explanations, classroom activities, feedback, and writing support (Lee et al., 2024; Chan and Hu, 2023). Universities have responded with policy statements, syllabus templates, workshops, shared resources, consultation services, and access guidance (Wang et al., 2024; Jin et al., 2025; An et al., 2025; Sutedjo et al., 2025). Faculty development links these institutional responses to classroom enactment.

Assessment and risk management concentrate the course-level challenge. GenAI can produce fluent texts, summaries, code, and feedback, so assessment needs evidence of student reasoning, process, and disciplinary performance (Xia et al., 2024; Foung et al., 2024). Policy analysis shows that originality and misconduct rules need translation into permitted support, disclosure requirements, process evidence, and criteria for inappropriate substitution (Luo, 2024; Tsao, 2025). Because GenAI outputs can contain factual errors, fabricated sources, bias, privacy exposure, and overreliance, faculty also need routines for output review and responsibility allocation. Multicultural evidence shows that GenAI benefits and academic integrity are interpreted through educational and cultural background (Yusuf et al., 2024; Al-khresheh, 2024).

### 2.4 Prompt design as pedagogical task formulation

Prompt design in educational settings is pedagogical task formulation. It translates learning objectives, student level, content boundaries, task constraints, assessment criteria, and responsibility rules into instructions that GenAI can process. Prompt engineering research for educators emphasizes role, goal, audience, topic, structure, and output constraints (Park and Choo, 2024; Qian, 2025); education-focused prompt research links prompt structure to question clarification, evidence requirements, feedback paths, and learning support (Walter, 2024). In faculty development, prompts provide observable evidence of how faculty formulate GenAI-mediated teaching tasks that are executable, assessable, revisable, and accountable.

### 2.5 Comparative digital education contexts and institutional mediation

Cross-national digital education research distinguishes national, institutional, classroom, and individual levels of analysis (Bray et al., 2007). In the GenAI context, national digitalization strategies and model ecosystems shape opportunity structures, while faculty encounter them through institutional training, local rules, tool access, multilingual resources, disciplinary communities, and perceived permission to experiment. University policy studies show variation in academic integrity, privacy, stakeholder communication, equitable access, faculty preparation, and continuous evaluation (Jin et al., 2025; An et al., 2025; McDonald et al., 2025; Sutedjo et al., 2025).

The China–Kazakhstan comparison examines this institutional mediation. China offers extensive digital education platforms, smart-education policy, and domestic GenAI ecosystems (Ministry of Education of the People's Republic of China, 2025). Kazakhstan offers active AI infrastructure development, human-capital programs, multilingual resources, university AI programs, and KazLLM development (Prime Minister of the Republic of Kazakhstan, 2024). The baseline survey and training evaluation focus on how these conditions are associated with faculty members' everyday instructional decisions.

## 3 Theoretical framework and research questions

The analytical framework combines three ideas. Situated professional learning explains why faculty development is examined through course tasks, assessment work, and reflection (Guskey, 2002; Desimone, 2009). Technology-integrated pedagogical judgement explains why GenAI pedagogical readiness includes technical understanding, pedagogical reasoning, content knowledge, assessment judgement, ethics, and responsible use (Mishra and Koehler, 2006; Redecker, 2017; Long and Magerko, 2020; Ng et al., 2021; Miao and Holmes, 2024). Institutional mediation explains why comparative differences are analyzed through exposure, support, permission, resources, and risk interpretation (Bray et al., 2007; Jin et al., 2025). Together, these ideas connect baseline readiness, training design, and prompt-level evidence.

This framework motivates four research questions:

1. How do faculty members in the Chinese and Kazakhstani participating universities differ in baseline GenAI pedagogical readiness across the seven measured dimensions?
2. To what extent are these baseline differences associated with prior GenAI use, recent AI-related training, multilingual resource access, institutional support, perceived permission, policy clarity, risk sensitivity, and disciplinary background?
3. How does structured GenAI prompt-task training compare with conventional GenAI-focused faculty development in readiness improvement and delayed retention?
4. How do prompt logs and GenAI use patterns reveal post-training faculty development needs?

## 4 Materials and methods

### 4.1 Research design

The study used a mixed-methods field design with three linked components: a baseline comparison, a quasi-experimental training evaluation, and prompt-level qualitative analysis. The analysis focuses on five participating universities, three in China and two in Kazakhstan, recruited through institutional collaboration with faculty development offices and departmental coordinators. The training component was treated as a quasi-experimental field design because participation reflected institutional scheduling, training capacity, existing faculty development programs, and voluntary sign-up (Shadish et al., 2002).

**Figure 1:**
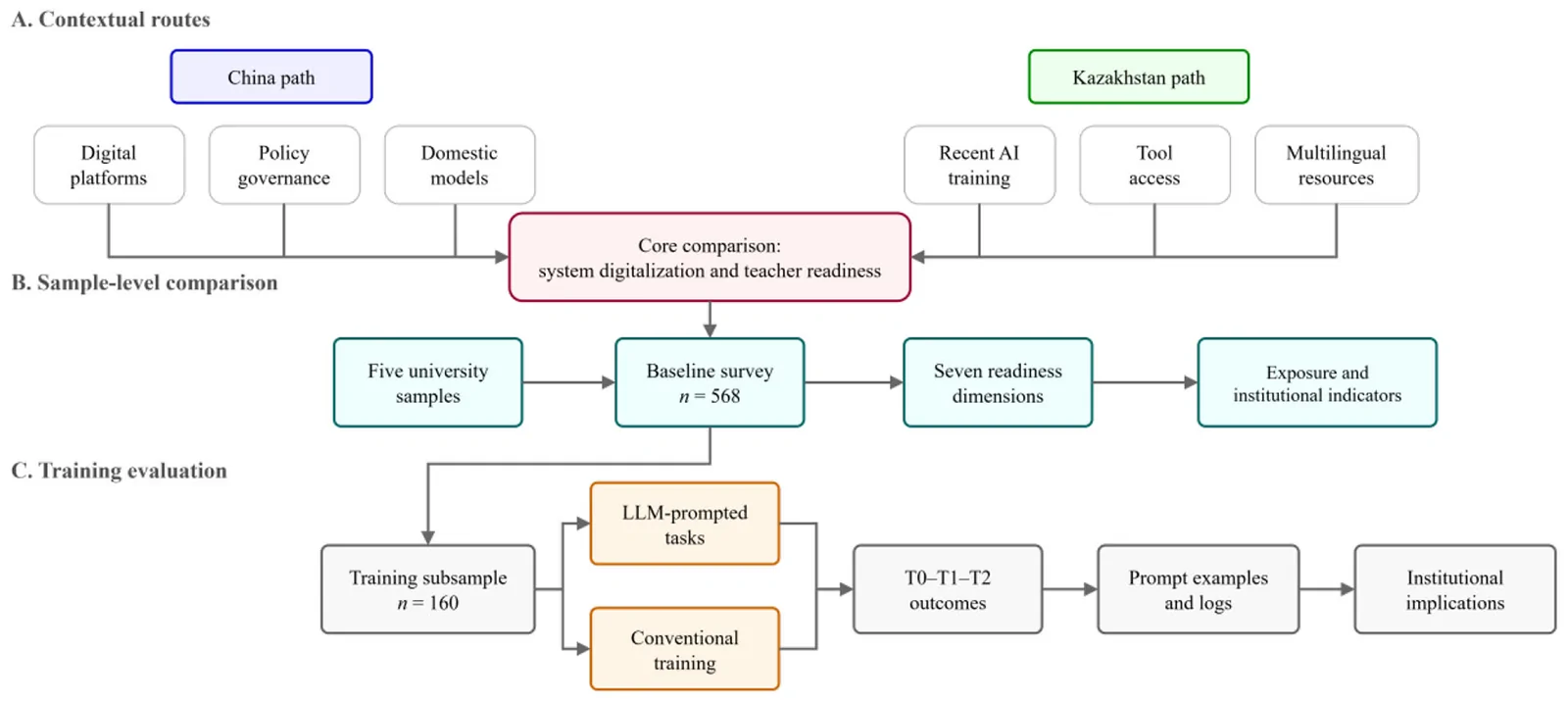
Research architecture for the empirical mixed-methods study.

The baseline component collected faculty background information and GenAI pedagogical readiness scores. The training component compared structured GenAI prompt-task training with conventional GenAI-focused faculty development. The qualitative component analyzed prompt examples and GenAI interaction records from the structured training, focusing on instructional-task formulation, output constraints, material revision, assessment criteria, and AI-use rules. Figure 1 summarizes the sequence of baseline survey, training, post-test, delayed test, and prompt-log analysis.

### 4.2 Participants and data sources

The baseline sample consisted of 568 university faculty members, including 340 from the three Chinese universities and 228 from the two Kazakhstani universities. Participants were recruited through faculty development offices, departmental announcements, and internal training communication channels. Eligibility required current higher education teaching responsibility, involvement in course design or classroom teaching, and completion of the baseline GenAI pedagogical readiness questionnaire. The sample covered STEM and applied disciplines, humanities and social sciences, and education or teacher-training programs.

The quasi-experimental training component included 160 faculty members drawn from the baseline sample: 80 in structured GenAI prompt-task training and 80 in conventional GenAI-focused faculty development. Faculty development offices and departmental coordinators organized registrants into workshop cohorts according to faculty availability, course schedules, site capacity, and the existing training calendar. Participants enrolled through the institutional training channel rather than selecting between two named research conditions, and each condition included faculty from both countries. Structured and conventional sessions were delivered separately, used separate task sheets and prompt-record forms, and participants were asked not to circulate session materials across cohorts during the four-week training period. The structured group focused on course-objective setting, prompt design, generated-content verification, teaching-material revision, assessment-criteria adjustment, and AI-use rule explanation. The conventional group received GenAI function introduction, teaching-case explanation, and general application advice as an active comparison condition.

The data sources included the baseline questionnaire, training-subsample pre-test (T0), immediate post-test (T1), delayed post-test (T2), prompt examples, GenAI interaction logs, usage-pattern summaries, and training satisfaction feedback. The baseline questionnaire recorded age, years of teaching experience, gender, discipline, prior weekly GenAI-use frequency, AI-related training in the previous six months, perceived institutional support, perceived permission to experiment, multilingual GenAI resource access, policy clarity, risk sensitivity, and perceived lack of training. Perceived indicators used a 1–5 agreement scale. Cases were screened for eligibility, duplicate submissions, incomplete readiness-scale responses, and implausible response patterns. The final baseline sample included 116, 113, and 111 faculty members from the three Chinese universities and 119 and 109 faculty members from the two Kazakhstani universities. The training subsample included 83 Chinese and 77 Kazakhstani faculty members. Both training groups completed T0, T1, and T2, yielding 0 longitudinal attrition and no readiness-scale missing data. Complete cases were used for analysis. Supplementary Table S1 provides the scale items and scoring used in the baseline questionnaire, and Supplementary Table S2 reports sample flow, university-level counts, attrition, and missing-data handling.

### 4.3 Competence framework and instruments

The GenAI pedagogical readiness scale was designed around course-practice tasks. Initial item domains were derived from TPACK, DigCompEdu, AI literacy, GenAI assessment, and faculty development literature (Mishra and Koehler, 2006; Redecker, 2017; Long and Magerko, 2020; Ng et al., 2021; Xia et al., 2024; Foung et al., 2024; Amundsen and Wilson, 2012). The research team mapped these domains to faculty tasks: understanding GenAI capabilities and limits, formulating prompts, revising teaching materials, redesigning assessment, checking output quality, explaining responsible use, and adapting GenAI work to disciplinary standards. Bilingual faculty development researchers reviewed the draft items for clarity, disciplinary transferability, and cross-language equivalence; this review refined technical wording, prompt-design overlap, and ethics items on disclosure and privacy.

The scale contained seven dimensions, each measured by four items on a 1–5 agreement scale: basic GenAI understanding, prompt design, AI-supported instructional design, AI-supported assessment, critical judgement, educational ethics, and disciplinary transfer. Dimension scores were calculated as the mean of the four corresponding items, with higher scores indicating stronger self-reported GenAI pedagogical readiness.

Chinese, English, Kazakh, and Russian versions were prepared through translation, back-translation, and reconciliation. Bilingual researchers checked terms such as “prompt,” “hallucination,” “disclosure,” and “disciplinary transfer” against the English working version. Reliability coefficients were good across dimensions, with total-sample Cronbach's alpha ranging from 0.822 to 0.889 and McDonald's omega ranging from 0.825 to 0.891. Supplementary Table S3 reports dimension-level reliability for the total, Chinese, and Kazakhstani samples. Multi-group CFA and measurement-invariance testing provided the main validity evidence. Supplementary Tables S4a, S4b report standardized factor loadings, partial scalar invariance details, latent-factor correlations, estimator, software, and missing-data treatment.

### 4.4 Training intervention

The structured GenAI prompt-task training organized GenAI use around course-material preparation, learning-task design, assessment revision, and AI-use rule explanation. The core cycle was prompt formulation, generated-output review, course-material revision, and written reflection. The intervention included four 90-min onsite workshops, delivered once per week for four consecutive weeks, for a total of 360 minutes. Training was conducted in computer classrooms or digital-teaching spaces at the five participating universities. The structured and conventional groups received the same number of sessions and comparable contact time, learning environment, facilitator support, and basic training materials.

Training tasks used DeepSeek-R1, displayed in the official web interface as “DeepThink (R1),” during the October 20–November 14, 2025 access period. The training used the web interface; API access, custom system prompts, and externally adjustable sampling parameters were not part of the protocol. Web search was off, and each task used a new conversation. Task records included access mode, access date and time, prompt text, generated response, faculty verification record, revised teaching material, and written reflection.

The structured training consisted of four course-work sessions. Session 1 rewrote a course objective into a prompt specifying objective, student level, prior knowledge, duration, language, and output format. Session 2 generated and revised a classroom activity. Session 3 generated or revised a rubric, added process evidence, and specified student disclosure of GenAI assistance. Session 4 identified hallucination, bias, privacy, and overreliance risks and drafted course-level AI-use rules. Each session yielded a prompt, a generated response, a faculty-revised teaching artifact, and a short reflection. Supplementary Table S5 provides the training protocol, and Supplementary Table S6 provides the structured prompt templates.

The structured prompt template required seven components: course objective, student level, task constraints, assessment criteria, faculty verification, student disclosure, and risk control. Faculty prompts and revised materials were evaluated with a performance checklist covering objective specificity, learner fit, constraint clarity, assessment alignment, output verification, responsible-use explanation, and disciplinary adaptation. Each participating university arranged one local facilitator, and two research-team members coordinated materials and implementation. Facilitators held at least a master's degree, had at least three years of higher education teaching, faculty development, or educational-technology training experience, and had prior experience with GenAI-supported teaching applications. Before formal training, facilitators attended a 120-min protocol briefing and a 60-min demonstration-task calibration. All sites used the same *GenAI Pedagogical Readiness Prompt-Task Training Manual, version 1.0* (GPR-PTT-v1.0), task sheets, prompt templates, performance checklist, and written reflection form. Participants wrote prompts in Chinese, Kazakh, Russian, or English according to course context; original-language prompts and outputs were retained, and English reporting versions were translated and checked by bilingual researchers.

The conventional GenAI-focused faculty development group served as an active comparison condition. It included GenAI function introduction, teaching-use examples, discussion of academic integrity and privacy concerns, and general suggestions for digital-teaching applications. It did not require the structured prompt-task cycle, faculty verification record, rubric revision, or course-level AI-use rule drafting.

### 4.5 Data analysis

The analysis followed the four research questions. Supplementary Table S7 documents the prompt-coding manual used for the qualitative analysis, and Supplementary Table S8 summarizes the estimability of university-level sensitivity checks. For RQ1, descriptive statistics, independent-sample comparisons, 95% confidence intervals for mean differences, and Cohen's *d* compared Chinese and Kazakhstani faculty on the seven baseline readiness dimensions. Measurement invariance was examined through configural, metric, scalar, and partial scalar models using changes in CFI and RMSEA (Meredith, 1993; Cheung and Rensvold, 2002; Chen, 2007).

For RQ2, hierarchical regression models predicted overall baseline GenAI pedagogical readiness. Country membership, demographic variables and disciplinary background, prior GenAI use and recent AI-related training, and institutional-contextual indicators were entered sequentially. Multicollinearity, residual patterns, and influential observations were checked with variance inflation factors, residual plots, standardized residuals, and leverage diagnostics. Because the data came from five universities, university-level sensitivity checks were treated as descriptive rather than confirmatory. Conventional cluster-robust inference was not used as primary evidence with so few clusters, and university fixed effects could not estimate the country coefficient because universities were nested within country.

For RQ3, training-group baseline equivalence was checked before post-test comparison. Pre-post change, post-test group differences, and delayed retention were examined with paired comparisons, between-group comparisons, and a mixed-effects model for the T0–T1–T2 prompt-design trajectory. Post-test group comparisons used ordinary two-group tests based on the displayed group means, standard deviations, and *n* = 80 per group; the reported *F* values equal *t*^2^ with df = 158, and partial $\eta_{p}^{2}$ was calculated as *F*/(*F*+*df*_error_). The prompt-design trajectory model included fixed effects for group, time, and group-by-time interaction, with participant-level random intercepts. Group was coded 0 = conventional GenAI-focused faculty development and 1 = structured GenAI prompt-task training; time was dummy-coded with T0 as the reference. Mixed-model parameters were estimated with statsmodels MixedLM, and omnibus group, time, and group-by-time tests used Type III tests from the corresponding repeated-measures design; partial $\eta_{p}^{2}$ was calculated from effect and error sums of squares. Effect sizes were reported using Cohen's *d* and partial $\eta_{p}^{2}$, with 95% confidence intervals for major mean differences. Robust or non-parametric alternatives were used as sensitivity checks where distributional assumptions were weak.

For RQ4, prompt examples were analyzed through a rule-based qualitative coding scheme informed by thematic-analysis principles (Braun and Clarke, 2006). The coding unit was a prompt or prompt-revision segment. Each unit received one strategy-type code and was eligible for multiple pedagogical theme codes. Strategy types included technical, practical, exploratory, narrative, and structured prompts; pedagogical themes included learning objectives, instructional activities, assessment design, critical thinking, practical constraints, structured prompting, pedagogical innovation, contextual application, learner difficulties, academic integrity, and risk control. A 30% subsample, comprising 96 prompts, was double-coded. Disagreements were resolved through coder discussion and final confirmation against the coding manual in Supplementary Table S7. Co-occurrence frequencies between prompt strategies and pedagogical themes formed the strategy–theme matrix, and illustrative examples were selected from the coded task records.

## 5 Results

### 5.1 Baseline differences in GenAI pedagogical readiness

RQ1 asked how faculty members in the Chinese and Kazakhstani participating universities differed in baseline GenAI pedagogical readiness. Table 1 presents the measurement-invariance results. The configural model showed good fit, χ^2^ = 812.34, df = 329, CFI = 0.957, TLI = 0.948, RMSEA = 0.041, SRMR = 0.045. Metric invariance was supported by a small fit change, ΔCFI = −0.004. The scalar model showed a modest decline in fit, and a partial scalar model reached acceptable fit after two item intercepts were freed, CFI = 0.951, RMSEA = 0.042, ΔCFI = −0.002.

**Table 1:** Measurement invariance across Chinese and Kazakhstani samples.

Model	*N*	χ^2^	df	CFI	TLI	RMSEA	SRMR	ΔCFI	Decision
Configural invariance	568	812.34	329	0.957	0.948	0.041	0.045	–	supported
Metric invariance	568	846.72	350	0.953	0.949	0.042	0.049	−0.004	supported
Scalar invariance	568	891.56	371	0.944	0.943	0.045	0.054	−0.009	acceptable
Partial scalar invariance	568	864.18	366	0.951	0.948	0.042	0.051	−0.002	supported

*N* = 340 faculty members from China and 228 from Kazakhstan. CFI, comparative fit index; TLI, Tucker–Lewis index; RMSEA, root mean square error of approximation; SRMR, standardized root mean square residual.

Table 2 presents the baseline comparison. The Kazakhstani sample reported higher scores on all seven dimensions. The clearest differences were in disciplinary transfer (*d*=-0.480), prompt design (*d*=-0.462), basic understanding (*d*=-0.433), and assessment (*d*=-0.406). Instructional design, critical judgement, and ethics showed smaller differences. The effect sizes were small to moderate and consistent across the seven dimensions.

**Table 2:** Cross-country baseline comparison of GenAI pedagogical readiness.

Dimension	China n	China M	China SD	KZ n	KZ M	KZ SD	Diff.	95% CI	*t*	*p*	*d*
C1: Basic understanding	340	2.604	0.568	228	2.845	0.546	−0.241	[-0.334, −0.148]	−5.031	< 0.001	−0.433
C2: Prompt design	340	2.196	0.540	228	2.443	0.529	−0.247	[-0.336, −0.158]	−5.375	< 0.001	−0.462
C3: Instructional design	340	2.803	0.573	228	2.974	0.542	−0.171	[-0.264, −0.078]	−3.548	< 0.001	−0.306
C4: Assessment	340	2.714	0.583	228	2.941	0.538	−0.228	[-0.320, −0.134]	−4.703	< 0.001	−0.406
C5: Critical judgement	340	2.695	0.583	228	2.858	0.538	−0.162	[-0.256, −0.070]	−3.348	< 0.001	−0.289
C6: Ethics	340	2.910	0.560	228	3.045	0.507	−0.135	[-0.224, −0.046]	−2.909	0.004	−0.252
C7: Disciplinary transfer	340	2.284	0.552	228	2.537	0.502	−0.254	[-0.341, −0.165]	−5.551	< 0.001	−0.480

KZ, Kazakhstan. Diff., China minus Kazakhstan mean difference. CI, confidence interval for the mean difference. Negative values indicate higher Kazakhstan baseline scores.

### 5.2 Exposure and institutional conditions explaining baseline differences

RQ2 examined whether baseline differences were associated with exposure and institutional conditions. Table 3 presents the contextual indicators. The two samples were similar in age, teaching experience, gender distribution, and disciplinary composition. The Kazakhstani sample reported higher prior weekly GenAI use, more recent AI-related training, stronger institutional support, greater perceived permission, and better multilingual GenAI resource access. The Chinese sample reported higher policy clarity, risk sensitivity, and perceived lack of training.

**Table 3:** Country-level descriptive differences in contextual indicators.

Variable	China n	China	KZ n	Kazakhstan	Test	*p*	Effect size
Age, M (SD)	340	39.6 (8.2)	228	38.8 (7.9)	*t* = 1.15	0.252	*d* = 0.10
Teaching years, M (SD)	340	12.4 (7.1)	228	11.6 (6.8)	*t* = 1.33	0.184	*d* = 0.11
Female, %	340	57.4	228	59.2	χ^2^ = 0.18	0.671	ϕ = 0.02
STEM / applied disciplines, %	340	38.5	228	41.7	χ^2^ = 0.58	0.446	ϕ = 0.03
Humanities / social sciences, %	340	44.1	228	39.9	χ^2^ = 0.98	0.322	ϕ = 0.04
Education / teacher training, %	340	17.4	228	18.4	χ^2^ = 0.09	0.764	ϕ = 0.01
Prior GenAI use at least weekly, %	340	34.7	228	49.1	χ^2^ = 11.78	< 0.001	ϕ = 0.14
AI-related training in past 6 months, %	340	27.9	228	56.6	χ^2^ = 46.83	< 0.001	ϕ = 0.29
Perceived institutional support, M (SD)	340	2.86 (0.82)	228	3.18 (0.79)	*t* = −4.62	< 0.001	*d*=-0.40
Perceived permission to experiment, M (SD)	340	2.71 (0.88)	228	3.27 (0.84)	*t* = −7.55	< 0.001	*d*=-0.65
Multilingual GenAI resource access, M (SD)	340	2.79 (0.91)	228	3.36 (0.86)	*t* = −7.49	< 0.001	*d*=-0.64
Policy clarity, M (SD)	340	3.15 (0.81)	228	2.96 (0.84)	*t* = 2.70	0.007	*d* = 0.23
Risk sensitivity, M (SD)	340	3.82 (0.76)	228	3.41 (0.79)	*t* = 6.19	< 0.001	*d* = 0.53
Perceived lack of training, M (SD)	340	3.48 (0.83)	228	3.02 (0.88)	*t* = 6.33	< 0.001	*d* = 0.54

KZ, Kazakhstan. Values are means and standard deviations unless otherwise indicated. Continuous variables use Cohen's *d*; categorical variables use ϕ.

Table 4 reports the hierarchical regression models. In the unadjusted model, the Kazakhstan coefficient was positive and statistically significant, *B* = 0.205, SE = 0.035, *p* < 0.001. The coefficient remained significant after demographic and discipline controls, *B* = 0.188, but decreased after prior GenAI use and recent AI-related training entered the model, *B* = 0.092. When institutional support, permission to experiment, multilingual resource access, policy clarity, and risk sensitivity were included, the coefficient decreased to *B* = 0.041 and was no longer statistically significant.

**Table 4:** Hierarchical regression explaining baseline GenAI pedagogical readiness.

Predictor	Model 1 B (SE)	Model 2 B (SE)	Model 3 B (SE)	Model 4 B (SE)
Kazakhstan sample	0.205 (0.035)***	0.188 (0.034)***	0.092 (0.038)*	0.041 (0.034)
Age	–	−0.003 (0.002)	−0.002 (0.002)	−0.001 (0.002)
Teaching years	–	0.004 (0.003)	0.003 (0.003)	0.002 (0.003)
STEM / applied discipline	–	0.071 (0.033)*	0.059 (0.031)	0.041 (0.030)
Education / teacher training discipline	–	0.064 (0.038)	0.052 (0.036)	0.044 (0.034)
Prior GenAI use frequency	–	–	0.121 (0.018)***	0.096 (0.017)***
AI-related training in past 6 months	–	–	0.148 (0.032)***	0.103 (0.030)***
Perceived institutional support	–	–	–	0.083 (0.018)***
Perceived permission to experiment	–	–	–	0.094 (0.017)***
Multilingual resource access	–	–	–	0.068 (0.017)***
Policy clarity	–	–	–	0.036 (0.017)*
Risk sensitivity	–	–	–	−0.052 (0.020)**
*R* ^2^	0.057	0.096	0.318	0.442
Δ*R*^2^	–	0.039	0.222	0.124
Country coefficient reduction	–	8.3%	55.1%	80.0%

*N* = 568. Outcome, overall baseline GenAI pedagogical readiness. Country coded China = 0, Kazakhstan = 1. SE, standard error. *p < 0.05. **p < 0.01. ***p < 0.001.

### 5.3 Readiness changes after structured GenAI prompt-task training

RQ3 compared structured GenAI prompt-task training with conventional GenAI-focused faculty development. Table 5 presents the baseline equivalence checks. The two groups showed no statistically significant differences in country composition, gender distribution, age, years of teaching experience, prior weekly GenAI use, recent AI-related training, institutional support, perceived permission, risk sensitivity, or T0 readiness dimensions.

**Table 5:** Baseline equivalence between training groups.

Variable	Structured group (*n* = 80)	Conventional group (*n* = 80)	Test	*p*	Effect size
China sample, %	52.5	51.3	χ^2^ = 0.02	0.873	ϕ = 0.01
Female, %	58.8	56.3	χ^2^ = 0.10	0.751	ϕ = 0.03
Age, M (SD)	39.2 (7.8)	38.7 (8.1)	*t* = 0.40	0.692	*d* = 0.06
Teaching years, M (SD)	11.8 (6.9)	11.4 (7.2)	*t* = 0.36	0.721	*d* = 0.06
Prior GenAI weekly use, %	41.3	38.8	χ^2^ = 0.10	0.748	ϕ = 0.03
AI-related training in past 6 months, %	36.3	33.8	χ^2^ = 0.11	0.742	ϕ = 0.03
Institutional support, M (SD)	3.02 (0.81)	2.96 (0.84)	*t* = 0.46	0.646	*d* = 0.07
Perceived permission, M (SD)	2.94 (0.86)	2.88 (0.90)	*t* = 0.43	0.670	*d* = 0.07
Risk sensitivity, M (SD)	3.61 (0.79)	3.67 (0.82)	*t*=-0.47	0.639	*d*=-0.07
C1 Basic understanding T0	2.714 (0.581)	2.691 (0.566)	*t* = 0.25	0.801	*d* = 0.04
C2 Prompt design T0	2.338 (0.536)	2.302 (0.542)	*t* = 0.42	0.675	*d* = 0.07
C3 Instructional design T0	2.792 (0.571)	2.771 (0.558)	*t* = 0.24	0.812	*d* = 0.04
C4 Assessment T0	2.704 (0.584)	2.681 (0.579)	*t* = 0.25	0.803	*d* = 0.04
C5 Critical judgement T0	2.778 (0.575)	2.741 (0.563)	*t* = 0.41	0.683	*d* = 0.06
C6 Ethics T0	2.913 (0.552)	2.886 (0.548)	*t* = 0.31	0.756	*d* = 0.05
C7 Disciplinary transfer T0	2.398 (0.531)	2.372 (0.520)	*t* = 0.31	0.758	*d* = 0.05

Structured group, structured GenAI prompt-task training; Conventional group, conventional GenAI-focused faculty development.

**Figure 2:**
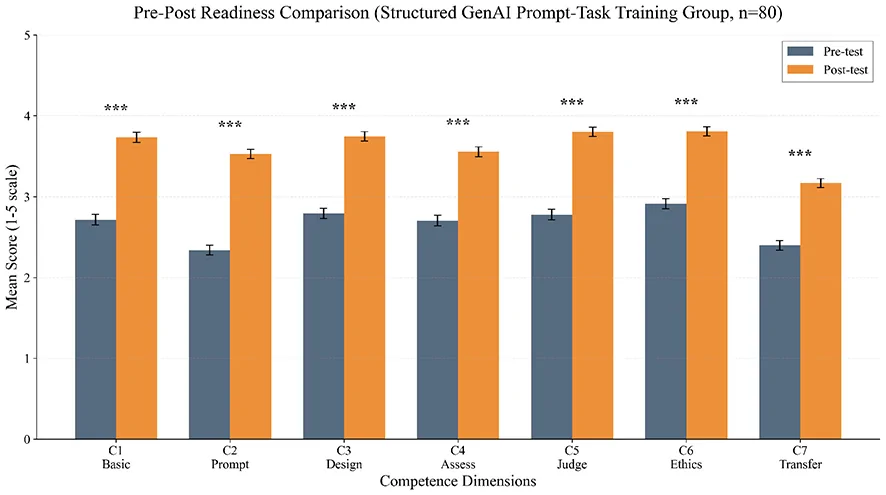
Pre-post comparison of GenAI pedagogical readiness in the structured GenAI prompt-task training group. Error bars represent standard errors.

As shown in Figure 2, faculty in the structured GenAI prompt-task training group showed post-training increases across all seven readiness dimensions. The largest gain was in prompt design, which rose from *M* = 2.338 to *M* = 3.527 (mean gain = 1.189). Basic understanding increased from *M* = 2.714 to *M* = 3.732, and critical judgement increased from *M* = 2.778 to *M* = 3.802. Disciplinary transfer rose from *M* = 2.398 to *M* = 3.168.

Table 6 presents the post-test group comparison based on ordinary two-group tests. The structured training group scored higher on all seven dimensions. The largest difference was in prompt design, with a mean difference of 1.049 points, *d* = 1.996, and partial $\eta_{p}^{2}=0.502$. Basic understanding, critical judgement, instructional design, ethics, disciplinary transfer, and assessment also showed large standardized differences.

**Table 6:** Post-test group comparison.

Dimension	Structured M	Structured SD	Conv. M	Conv. SD	Diff.	95% CI	*F*	*p*	*d*	$\eta_{p}^{2}$
C1: Basic understanding	3.732	0.573	2.863	0.529	0.869	[0.697, 1.041]	99.336	< 0.001	1.576	0.386
C2: Prompt design	3.527	0.503	2.478	0.547	1.049	[0.885, 1.213]	159.415	< 0.001	1.996	0.502
C3: Instructional design	3.746	0.531	2.993	0.539	0.753	[0.586, 0.920]	79.235	< 0.001	1.407	0.334
C4: Assessment	3.555	0.541	2.994	0.534	0.561	[0.393, 0.729]	43.572	< 0.001	1.044	0.216
C5: Critical judgement	3.802	0.514	2.934	0.558	0.868	[0.700, 1.036]	104.722	< 0.001	1.618	0.399
C6: Ethics	3.808	0.486	3.131	0.488	0.677	[0.525, 0.829]	77.300	< 0.001	1.390	0.329
C7: Disciplinary transfer	3.168	0.483	2.592	0.489	0.576	[0.424, 0.728]	56.184	< 0.001	1.185	0.262

Structured group *n* = 80; conventional group *n* = 80. Conv., conventional GenAI-focused faculty development group. Diff., structured minus conventional mean difference. CI, confidence interval for the mean difference. *F* = *t*^2^ from ordinary two-group comparisons with df = 158. Partial $\eta_{p}^{2}=F/\left(F+df_{\text{error}}\right)$.

Table 7 presents the linear mixed-effects model for the T0–T1–T2 prompt-design trajectory. The fixed-effect coefficient for structured group represents the baseline T0 group contrast, which was small and not statistically significant. The omnibus group effect in Panel B tests the average between-group difference across the three time points, and the group-by-time term tests whether the two trajectories differed over time. The model identified a significant group-by-time interaction, *F*(2, 316) = 220.45, *p* < 0.001, partial $\eta_{p}^{2}=0.582$. In the structured group, the T0-to-T1 gain was large, mean difference = 1.189, *p* < 0.001, *d* = 2.18, and the T0-to-T2 difference remained large, mean difference = 1.107, *p* < 0.001, *d* = 2.03. In the conventional group, the T0-to-T1 increase was modest, mean difference = 0.176, *p* = 0.006, *d* = 0.32, and the T2 score did not remain above baseline.

**Table 7:** Linear mixed-effects model for C2 prompt design across T0, T1, and T2.

Panel	Term/comparison	Estimate/mean	SE	*t*/*F*	df1	df2	*p*/effect size
A. Fixed effects	Intercept	2.302	0.061	37.74	–	–	< 0.001
	Structured group	0.036	0.086	0.42	–	–	0.675
	T1	0.176	0.064	2.75	–	–	0.006
	T2	−0.056	0.064	−0.88	–	–	0.379
	Structured group × T1	1.013	0.091	11.13	–	–	< 0.001
	Structured group × T2	1.163	0.091	12.78	–	–	< 0.001
B. Omnibus Type III tests	Group	–	–	148.92	1	158	< 0.001, $\eta_{p}^{2}=0.485$
	Time	–	–	412.36	2	316	< 0.001, $\eta_{p}^{2}=0.723$
	Group × time	–	–	220.45	2	316	< 0.001, $\eta_{p}^{2}=0.582$
C. Estimated means	Structured training, T0	2.338	–	–	–	–	–
	Structured training, T1	3.527	–	–	–	–	–
	Structured training, T2	3.445	–	–	–	–	–
	Conventional comparison, T0	2.302	–	–	–	–	–
	Conventional comparison, T1	2.478	–	–	–	–	–
	Conventional comparison, T2	2.246	–	–	–	–	–
D. Pairwise contrasts	Structured T1–T0	1.189	0.071	–	–	–	< 0.001, *d* = 2.18
	Structured T2–T0	1.107	0.073	–	–	–	< 0.001, *d* = 2.03
	Structured T2–T1	−0.082	0.040	–	–	–	0.042, *d*=-0.16
	Comparison T1–T0	0.176	0.064	–	–	–	0.006, *d* = 0.32
	Comparison T2–T0	−0.056	0.065	–	–	–	0.388, *d*=-0.10
	Comparison T2–T1	−0.232	0.067	–	–	–	0.001, *d*=-0.43

Group was coded 0 = conventional GenAI-focused faculty development and 1 = structured GenAI prompt-task training. Time was dummy-coded with T0 as the reference. The mixed model included fixed effects for group, time, and group-by-time interaction and a participant-level random intercept. Fixed-effect coefficients therefore describe baseline and time-referenced contrasts, whereas Panel B reports omnibus Type III tests for group, time, and group-by-time effects. Mixed-model parameters were estimated with statsmodels MixedLM; partial $\eta_{p}^{2}$ was calculated as *SS*_effect_/(*SS*_effect_+*SS*_error_) from the corresponding repeated-measures design.

**Figure 3:**
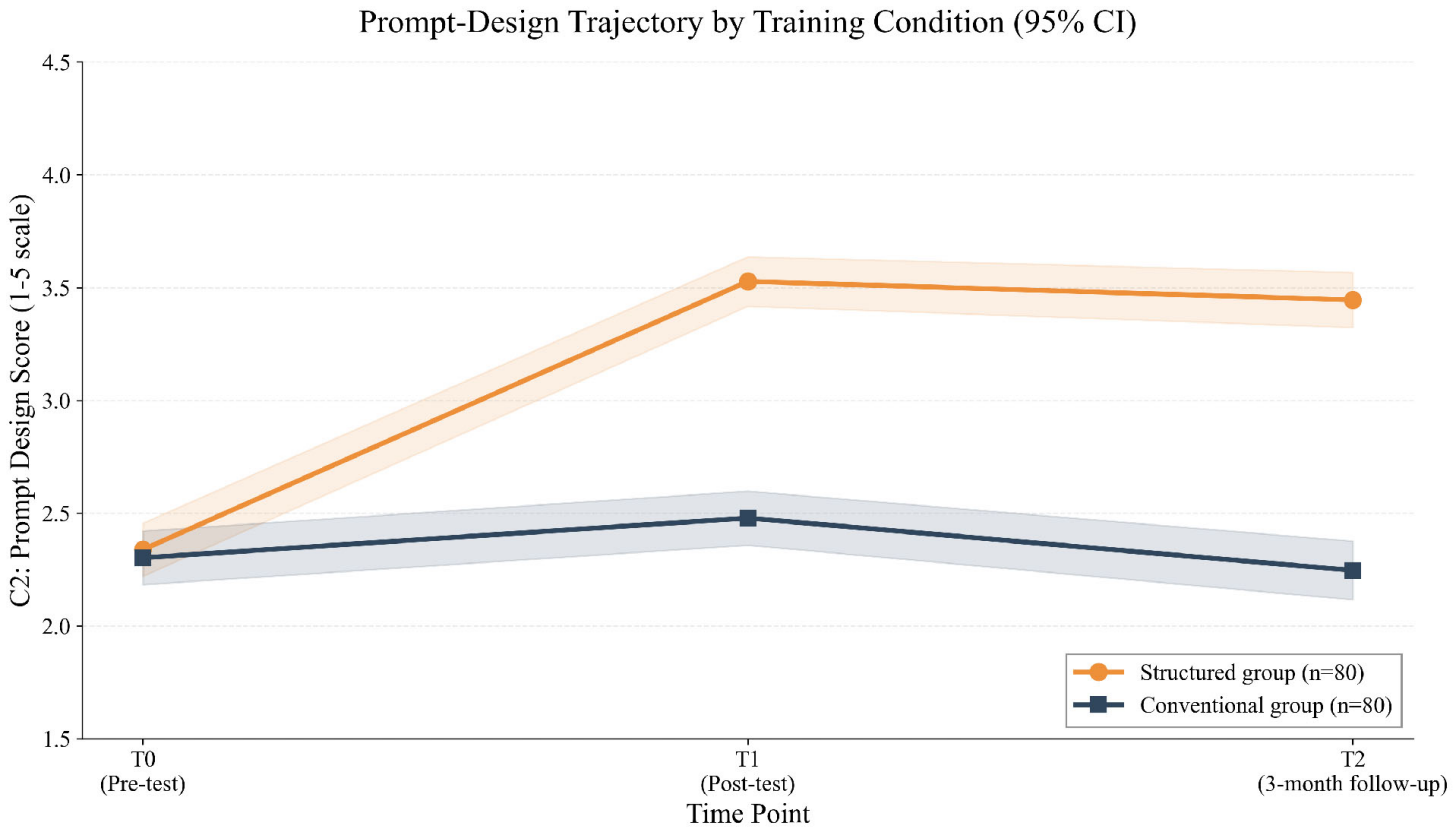
Prompt-design scores over time for the structured group and conventional group. Shaded areas represent 95% confidence intervals.

Figure 3 shows the corresponding time trajectory. The structured group had higher T1 prompt-design scores and retained much of that difference at T2, whereas the conventional group followed a flatter pattern.

### 5.4 Prompt evidence and post-training faculty development needs

**Figure 4:**
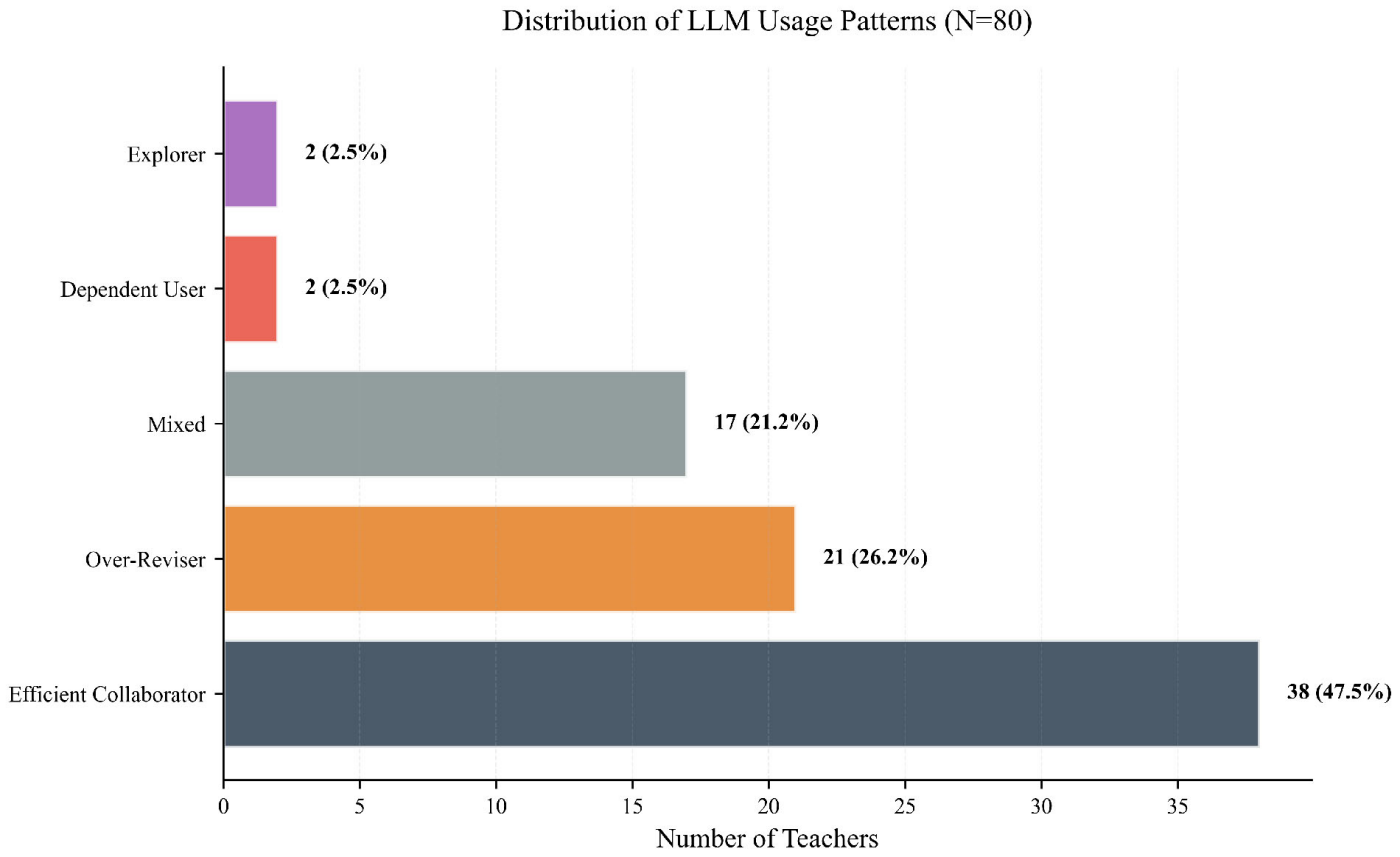
Distribution of GenAI use patterns in the structured GenAI prompt-task training group. The figure separates efficient collaboration, over-revision, mixed use, dependent use, and exploratory use as distinct post-training profiles.

RQ4 examined prompt logs and GenAI use patterns as evidence of post-training faculty development needs. Figure 4 shows the distribution of use patterns in the structured group. Efficient collaborators formed the largest category, accounting for 38 faculty members (47.5%). Over-revisers accounted for 21 faculty members (26.2%), and mixed users accounted for 17 (21.2%). Dependent users and explorers were rare, with two faculty members in each group. Efficient collaborators had a moderate revision rate (0.584) and relatively high satisfaction (3.737), whereas over-revisers had a higher revision rate (0.833).

Table 8 reports training satisfaction in the structured GenAI prompt-task training group. Continued use intention was the highest-rated dimension (*M* = 3.450), followed by training load (*M* = 3.438) and content relevance (*M* = 3.238). Tool usability was the lowest-rated dimension (*M* = 2.863).

**Table 8:** Training satisfaction in the structured GenAI prompt-task training group.

Dimension	Mean	SD	SE	95% CI lower	95% CI upper	N
Content relevance	3.238	0.945	0.106	3.030	3.445	80
Practical applicability	3.163	0.818	0.091	2.983	3.342	80
Tool usability	2.863	0.924	0.103	2.660	3.065	80
Training load	3.438	0.809	0.090	3.260	3.615	80
Continued use intention	3.450	0.899	0.100	3.253	3.647	80

Scores were measured on a 1–5 scale. Higher scores indicate more positive evaluation, except where scale interpretation depends on item wording.

Table 9 reports prompt-coding reliability. A 30% double-coded sample, consisting of 96 prompts, was used to examine agreement for prompt strategy type and pedagogical theme presence. Cohen's κ was 0.82 for prompt strategy type and 0.79 for pedagogical theme presence. For binary themes of learning objectives, assessment design, practical constraints, and learner difficulties, κ values ranged from 0.74 to 0.84. Overall coding agreement was 87.5%.

**Table 9:** Prompt coding reliability.

Coding category	Unit	Double-coded sample	Reliability statistic	Value
Prompt strategy type	prompt	96 prompts, 30% sample	Cohen's κ	0.82
Pedagogical theme presence	theme code	96 prompts, 30% sample	Cohen's κ	0.79
Learning objectives	binary theme	96 prompts	Cohen's κ	0.84
Assessment design	binary theme	96 prompts	Cohen's κ	0.81
Practical constraints	binary theme	96 prompts	Cohen's κ	0.76
Learner difficulties	binary theme	96 prompts	Cohen's κ	0.74
Overall coding agreement	all codes	96 prompts	Percent agreement	87.5%

Table 10 presents representative coded prompt examples. Technical prompts centered on lesson plans, steps, assessment criteria, and expected student outputs. Practical prompts focused on rubrics, process evidence, and academic integrity. Exploratory prompts opened alternative instructional designs. Narrative prompts used classroom scenarios to present ethical issues or learner difficulties. Structured prompts used fixed formats to align objectives, AI tasks, faculty checks, student outputs, and risk control.

**Table 10:** Illustrative coded prompt examples.

Strategy type	Prompt excerpt	Theme codes	Faculty revision focus	Interpretation
Technical	Generate a 45-minute lesson plan with objectives, steps, assessment criteria, and expected student outputs.	Learning objectives; instructional activities; assessment design	Added course-specific examples and timing	Foregrounds structure and measurable outcomes
Practical	Revise this rubric so that students must show how they used GenAI and what they changed manually.	Assessment design; academic integrity	Added process evidence and disclosure requirement	Connects GenAI use to assessable evidence
Exploratory	Give three possible ways to use GenAI to help students compare different explanations of the same concept.	Pedagogical innovation; critical thinking	Selected one option and added student discussion prompts	Opens alternatives that require classroom conversion
Narrative	Create a classroom scenario where students debate whether GenAI feedback should be accepted.	Contextual application; learner difficulties; ethics	Added discipline-specific evidence standard	Uses situated examples to discuss judgement
Structured	Use the following format: objective, AI task, faculty check, student output, risk control.	Structured prompting; practical constraints; risk control	Added verification steps and excluded personal data	Makes evaluation and risk management explicit
Structured	Draft a student-facing rule explaining when GenAI is allowed, when disclosure is required, and when use is prohibited.	Academic integrity; policy clarity; student disclosure	Rewrote rule into permitted, disclosed, prohibited categories	Translates policy into course language
Practical	Check this generated case for factual errors, biased assumptions, and mismatch with first-year students' prior knowledge.	Critical judgement; learner difficulties; disciplinary fit	Marked unsupported claims and simplified terminology	Shows verification before classroom use
Technical	Create feedback comments for three levels of student performance, but cite the rubric criteria and avoid adding new claims.	Assessment design; faculty verification; constraints	Removed unsupported feedback and tied comments to criteria	Connects feedback generation with assessment standards

**Figure 5:**
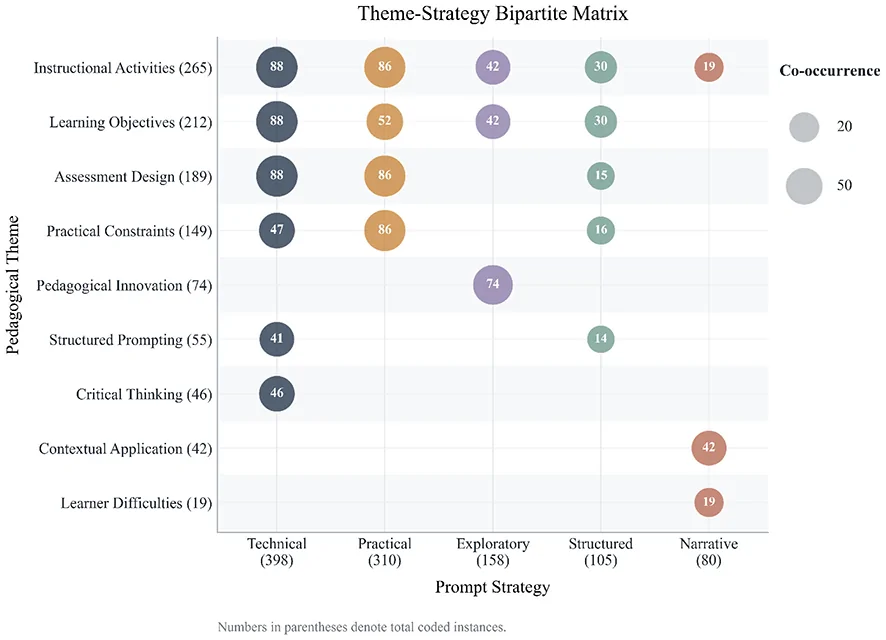
Theme–strategy bipartite matrix. Circle size indicates coded co-occurrence frequency. The matrix shows how different prompting strategies direct attention toward different pedagogical themes; numbers in parentheses indicate total coded instances.

Figure 5 presents the theme–strategy bipartite matrix. The matrix recorded 1,051 coded co-occurrences. Technical prompts accounted for the largest strategy total, with 398 instances, followed by practical prompts with 310, exploratory prompts with 158, structured prompts with 105, and narrative prompts with 80. By theme, instructional activities appeared most frequently, with 265 instances, followed by learning objectives with 212 and assessment design with 189. Technical prompts were mainly connected with learning objectives, instructional activities, assessment design, structured prompting, and critical thinking. Practical prompts were more strongly linked to assessment design, instructional activities, and practical constraints. Exploratory prompts concentrated in pedagogical innovation; narrative prompts were more often connected with contextual application and learner difficulties; structured prompts were distributed across instructional activities, learning objectives, assessment design, practical constraints, and structured prompting.

Prompt strategies differed in the pedagogical themes they foregrounded. Technical prompts supported lesson-plan structure and measurable outputs. Practical prompts connected assessment and rules. Exploratory prompts generated alternatives for instructional design. Narrative prompts represented ethical issues and learner difficulties in situated form. Structured prompts made objective specification, constraint setting, assessment criteria, faculty verification, student disclosure, and risk control explicit.

## 6 Discussion

The cross-country baseline pattern is best explained through the institutional conditions that connect national digital education agendas with faculty course practice. The Kazakhstani sample reported higher GenAI pedagogical readiness across the measured dimensions, with the clearest differences in prompt design, disciplinary transfer, and basic understanding. The hierarchical regression then showed that the Kazakhstan coefficient decreased substantially after prior GenAI use, recent AI-related training, institutional support, perceived permission to experiment, multilingual resource access, policy clarity, and risk sensitivity were entered into the model. This pattern directs attention away from country labels as stand-alone explanations and toward the channels through which faculty encounter GenAI in their work: access to tools and language resources, recent opportunities to practice with AI, local permission structures, policy signals, and risk interpretation. Comparative education research treats national context as one layer in a multi-level structure (Bray et al., 2007), and GenAI policy studies similarly show that guidelines, workshops, access arrangements, consultation resources, and responsibility-allocation mechanisms mediate how AI enters teaching (Wang et al., 2024; Jin et al., 2025; An et al., 2025; McDonald et al., 2025; Sutedjo et al., 2025). The results therefore indicate that institutional conditions matter when they become actionable in course design, assessment planning, disclosure rules, output verification, and discipline-specific adaptation. Research on educators' AI literacy and adoption supports this interpretation by linking AI use to professional development, technical experience, perceived usefulness, self-efficacy, and organizational support (Ayyoub et al., 2025; Mah and Groß, 2024; Bhat et al., 2024; Granström and Oppi, 2025).

The training findings link structured GenAI prompt-task training with readiness gains beyond those observed in conventional GenAI-focused faculty development. Faculty participating in the structured condition showed higher post-test readiness, especially in prompt design and critical judgement, and prompt-design gains remained visible at delayed testing. This pattern fits the intervention cycle: faculty began with learning objectives, converted them into prompts, reviewed generated outputs, revised teaching materials, and wrote reflections on the fit between AI-generated content and course needs. Across these tasks, GenAI use was embedded in instructional decision-making. Goal setting clarified what the output had to serve; prompt construction translated pedagogical intent into executable instructions; output checking required faculty to judge accuracy, bias, learner fit, and assessment alignment; material revision linked the generated text back to the course; written reflection turned the task into professional learning evidence. Reviews and case studies of GenAI implementation emphasize clear pedagogical frameworks, assessment tasks, and responsibility boundaries (Belkina et al., 2025; Li et al., 2025), and the present training results show how such frameworks operate inside a faculty development protocol. The practical lesson is that faculty development centers can support prompt design, critical judgement, instructional design, and assessment capacity by asking faculty to work on their own course artifacts under a structured prompt-task cycle.

The prompt logs clarify the theoretical status of prompt design in GenAI-supported teaching. Prompt design is not a detached technical skill; it is a form of course-level teaching-task design and professional judgement. The coded prompts showed faculty specifying learning objectives, student level, output constraints, assessment criteria, faculty verification, student disclosure, and risk control. These elements jointly define whether a GenAI-mediated task is executable, assessable, revisable, and accountable. TPACK explains why the prompt must connect technology, pedagogy, and content knowledge (Mishra and Koehler, 2006); AI literacy explains why faculty need to understand system capabilities, evaluate outputs, and make responsible decisions (Long and Magerko, 2020); intelligent-TPACK and education-focused prompt research further connect technical capability with pedagogical reasoning, disciplinary standards, feedback paths, and evidence requirements (Celik, 2023; Walter, 2024). The prompt evidence extends these perspectives by locating the integration point in concrete course instructions: objectives and student level define the learning problem, constraints and criteria define acceptable output, verification defines faculty responsibility, disclosure defines student-facing accountability, and risk control defines the boundary between useful assistance and unsafe adoption. This interpretation also aligns with research showing that faculty anxiety, trust, and policy interpretation shape GenAI use in practice (Viberg et al., 2025; Verano-Tacoronte et al., 2025; Tsao, 2025), because those concerns become operational through output review, assessment evidence, and AI-use rules.

The use-pattern evidence turns the discussion toward differentiated faculty development support. Efficient collaborators used GenAI with revision routines that can be extended to more complex course tasks; over-revisers need criteria for deciding when a teaching artifact is ready for use; mixed users need support in stabilizing their workflow across task types; dependent users need stronger routines for verification and faculty responsibility; exploratory users need help converting ideas into assessable classroom steps. These profiles show why a single GenAI workshop design serves faculty unevenly. Prompt records identify how faculty formulate objectives, constraints, and verification requests; revision behavior shows whether they accept, overwork, or redirect generated outputs; satisfaction feedback identifies friction in usability, workload, and perceived relevance. Faculty development centers can use this evidence to organize support around training design, course-material checking, assessment reconstruction, AI-use rules, and disciplinary transfer. Such support keeps GenAI training close to the work faculty actually perform: designing learning tasks, judging generated content, revising materials, explaining acceptable AI use to students, and adapting tools to disciplinary standards.

## 7 Limitations and future research

This study draws on data from five universities in China and Kazakhstan and primarily uses a self-report scale to examine faculty members' GenAI pedagogical readiness and the institutional support conditions surrounding their work. The questionnaire was prepared through multilingual translation, back-translation, and reconciliation, and measurement-invariance testing supported cross-sample comparison. Concept interpretation and response styles across language backgrounds may still have affected part of the evidence, especially for concepts such as AI literacy, ethical reasoning, prompt design, and disciplinary transfer (Vieluf et al., 2013; Viberg et al., 2025). The training subsample used a quasi-experimental design in which group assignment reflected voluntary faculty sign-up, institutional training arrangements, and course schedules. The training results are therefore best interpreted as associations between structured GenAI prompt-task training and changes in readiness rather than as strict causal estimates. The five participating universities capture practice differences under varied institutional and resource conditions, while broader samples are needed to cover the full range of university types and disciplinary contexts in the two countries.

Future research can expand the university and disciplinary samples and combine course materials, classroom observations, prompt records, and student learning outcomes to examine whether GenAI pedagogical readiness translates into enacted teaching practice. Randomized or matched designs, comparisons across different GenAI tools, and longer follow-up periods would further test training effects and their durability.

## 8 Conclusion

This study examined GenAI pedagogical readiness in five Chinese and Kazakhstani universities through baseline comparison, quasi-experimental training evaluation, and prompt-level analysis. Baseline readiness differences were associated with GenAI exposure, recent training, multilingual resource access, institutional support, perceived permission, policy clarity, risk sensitivity, and disciplinary background. Faculty participating in structured GenAI prompt-task training showed larger readiness gains than those participating in conventional GenAI-focused faculty development, and prompt logs showed how readiness is enacted through objective specification, output verification, assessment revision, AI-use rule explanation, and disciplinary transfer. The study positions GenAI faculty development as a course-level professional learning task in which training design, institutional support, and accountable prompt design work together.

## Data Availability

The raw data supporting the conclusions of this article will be made available by the authors, without undue reservation.
