# Supplementary material for: Research on the optimization of the training system of university faculty development centers in the context of GenAI: a comparative analysis based on Chinese and Kazakhstani universities
Source: Front Psychol. 2026 Sep 9;17:1890508. doi: 10.3389/fpsyg.2026.1890508 (PMC13599494; doi:10.3389/fpsyg.2026.1890508)
Supplement: Supplementary file 1 [file Data_Sheet_1.pdf]

# Supplementary Material

## 1 SUPPLEMENTARY TABLES

### 1.1 Supplementary Table S1. GenAI pedagogical readiness scale

Table S1: GenAI pedagogical readiness scale: dimensions, items, source basis, language versions, and scoring.

| Code | Dimension | Item wording used for documentation | Source or adaptation basis | Score |
|---|---|---|---|---|
| C1.1 | Basic GenAI understanding | I can explain common educational uses of GenAI in higher education courses. | AI literacy; DigCompEdu | 1–5 |
| C1.2 | Basic GenAI understanding | I can explain the main limitations of GenAI outputs when they are used in my course. | AI literacy; GenAI education risks | 1–5 |
| C1.3 | Basic GenAI understanding | I can distinguish appropriate GenAI assistance from uses that may weaken student learning evidence. | GenAI assessment; academic integrity | 1–5 |
| C1.4 | Basic GenAI understanding | I can identify teaching tasks in my course where GenAI may or may not be useful. | TPACK; faculty development | 1–5 |
| C2.1 | Prompt design | I can write prompts that specify learning objectives, student level, output format, and constraints. | Prompt engineering; TPACK | 1–5 |
| C2.2 | Prompt design | I can revise a prompt when the generated output is too general, inaccurate, or unsuitable for students. | Prompt revision; AI literacy | 1–5 |
| C2.3 | Prompt design | I can include assessment criteria or quality standards in prompts for teaching tasks. | GenAI assessment; prompt design | 1–5 |
| C2.4 | Prompt design | I can write prompts that require GenAI to show assumptions, limitations, or alternative options. | Critical judgement; prompt design | 1–5 |
| C3.1 | AI-supported instructional design | I can use GenAI to draft classroom activities aligned with course objectives. | TPACK; DigCompEdu | 1–5 |
| C3.2 | AI-supported instructional design | I can adapt generated teaching materials to students' prior knowledge and learning difficulties. | Faculty development; learner support | 1–5 |
| C3.3 | AI-supported instructional design | I can revise generated examples, cases, or explanations to fit my disciplinary context. | TPACK; disciplinary transfer | 1–5 |
| C3.4 | AI-supported instructional design | I can decide when generated materials should be rejected or substantially rewritten before classroom use. | AI literacy; critical judgement | 1–5 |

| Code | Dimension | Item wording used for documentation | Source or adaptation basis | Score |
|---|---|---|---|---|
| C4.1 | AI-supported assessment | I can use GenAI to draft assessment rubrics that align with learning outcomes. | GenAI assessment | 1–5 |
| C4.2 | AI-supported assessment | I can revise assignments so that students provide evidence of process, reasoning, and GenAI disclosure. | GenAI assessment; academic integrity | 1–5 |
| C4.3 | AI-supported assessment | I can evaluate whether generated feedback is consistent with rubric criteria and student work. | Assessment literacy; GenAI feedback | 1–5 |
| C4.4 | AI-supported assessment | I can explain to students which GenAI-supported assessment practices are permitted or prohibited. | Policy enactment; academic integrity | 1–5 |
| C5.1 | Critical judgement | I can check generated content for factual errors, fabricated references, and unsupported claims. | AI literacy; hallucination risk | 1–5 |
| C5.2 | Critical judgement | I can detect bias, inappropriate assumptions, or unfair learner representations in generated materials. | AI ethics; responsible use | 1–5 |
| C5.3 | Critical judgement | I can judge whether generated content fits the disciplinary standards of my course. | TPACK; disciplinary judgement | 1–5 |
| C5.4 | Critical judgement | I can decide what must be verified by the teacher before generated content is used with students. | Faculty verification; GenAI risk | 1–5 |
| C6.1 | Educational ethics | I can explain privacy risks related to entering course, student, or assessment information into GenAI tools. | AI ethics; policy guidance | 1–5 |
| C6.2 | Educational ethics | I can design student disclosure rules for GenAI assistance in my course. | Academic integrity; GenAI policy | 1–5 |
| C6.3 | Educational ethics | I can discuss fairness and access issues when students use GenAI unevenly. | Equity; responsible use | 1–5 |
| C6.4 | Educational ethics | I can balance pedagogical benefit with academic integrity when planning GenAI-supported activities. | GenAI assessment; ethics | 1–5 |
| C7.1 | Disciplinary transfer | I can adapt GenAI-supported tasks to the epistemic standards of my discipline. | TPACK; disciplinary transfer | 1–5 |
| C7.2 | Disciplinary transfer | I can judge whether a GenAI output uses disciplinary terminology accurately. | Critical judgement; content knowledge | 1–5 |
| C7.3 | Disciplinary transfer | I can design GenAI-supported tasks that match the learning products expected in my field. | TPACK; assessment design | 1–5 |
| C7.4 | Disciplinary transfer | I can explain discipline-specific boundaries for acceptable student GenAI use. | Academic integrity; disciplinary norms | 1–5 |

*Note.* Language versions: Chinese, English, Kazakh, and Russian. The English wording above is the documentation version used for manuscript reporting. Scale: 1 = strongly disagree, 5 = strongly agree.

## 1.2 Supplementary Table S2. Sample flow, attrition, and missing-data handling

Table S2: Sample flow, attrition, and missing-data handling.

| Stage / source | China | Kazakhstan | Total |
|---|---|---|---|
| Eligible baseline responses after screening | 340 | 228 | 568 |
| Chinese university 1 | 116 | – | 116 |
| Chinese university 2 | 113 | – | 113 |
| Chinese university 3 | 111 | – | 111 |
| Kazakhstani university 1 | – | 119 | 119 |
| Kazakhstani university 2 | – | 109 | 109 |
| Training subsample at T0 | 83 | 77 | 160 |
| Structured training group at T0 | 42 | 38 | 80 |
| Structured training group at T1 | 42 | 38 | 80 |
| Structured training group at T2 | 42 | 38 | 80 |
| Conventional training group at T0 | 41 | 39 | 80 |
| Conventional training group at T1 | 41 | 39 | 80 |
| Conventional training group at T2 | 41 | 39 | 80 |

*Note.* Both training groups completed the T0, T1, and T2 measurements. Longitudinal attrition was 0 participants, corresponding to an attrition rate of 0.00%. No attrition reasons such as course-schedule conflict, withdrawal, or incomplete delayed testing were recorded. The 568 eligible baseline questionnaires generated 15,904 item-level observations across the 28 readiness items, with 0 missing item responses and an item-missing rate of 0.00%. The training subsample had no readiness-scale missing data at T0, T1, or T2. During screening, duplicate submissions, ineligible cases, incomplete readiness-scale responses, and implausible response patterns were excluded. The final analysis retained only cases with complete responses to all 28 readiness items; mean substitution, multiple imputation, and other missing-value estimation methods were not used. Dimension scores were computed as the arithmetic mean of the four corresponding items. Regression models, CFA, measurement-invariance testing, and longitudinal comparisons used complete-case analysis.

## 1.3 Supplementary Table S3. Dimension reliability

Table S3: Internal consistency for the seven readiness dimensions.

| Dimension | Items | Total alpha | Total omega | China alpha | Kazakhstan alpha |
|---|---|---|---|---|---|
| Basic GenAI understanding | 4 | 0.844 | 0.846 | 0.838 | 0.851 |
| Prompt design | 4 | 0.878 | 0.880 | 0.872 | 0.885 |
| AI-supported instructional design | 4 | 0.856 | 0.858 | 0.849 | 0.864 |
| AI-supported assessment | 4 | 0.848 | 0.851 | 0.841 | 0.857 |

| Dimension | Items | Total alpha | Total omega | China alpha | Kazakhstan alpha |
|---|---|---|---|---|---|
| Critical judgement | 4 | 0.889 | 0.891 | 0.883 | 0.896 |
| Educational ethics | 4 | 0.822 | 0.825 | 0.816 | 0.831 |
| Disciplinary transfer | 4 | 0.867 | 0.869 | 0.861 | 0.875 |

*Note.* Alpha = Cronbach's alpha; omega = McDonald's omega. All coefficients were calculated from baseline item-level data. The seven dimensions all exceeded .80 for both alpha and omega, indicating good internal consistency. Reliability coefficients were similar across the Chinese and Kazakhstani samples.

## 1.4 Supplementary Table S4. CFA loadings, partial scalar invariance, and inter-dimension correlations

### 1.4.1 Supplementary Table S4a. Standardized factor loadings

Table S4a: Standardized CFA factor loadings.

| Dimension | Item | Standardized loading | SE |
|---|---|---|---|
| Basic GenAI understanding | C1.1 | 0.740 | 0.031 |
| Basic GenAI understanding | C1.2 | 0.790 | 0.028 |
| Basic GenAI understanding | C1.3 | 0.810 | 0.027 |
| Basic GenAI understanding | C1.4 | 0.760 | 0.030 |
| Prompt design | C2.1 | 0.830 | 0.025 |
| Prompt design | C2.2 | 0.860 | 0.023 |
| Prompt design | C2.3 | 0.800 | 0.027 |
| Prompt design | C2.4 | 0.780 | 0.029 |
| AI-supported instructional design | C3.1 | 0.790 | 0.028 |
| AI-supported instructional design | C3.2 | 0.820 | 0.026 |
| AI-supported instructional design | C3.3 | 0.770 | 0.030 |
| AI-supported instructional design | C3.4 | 0.810 | 0.027 |
| AI-supported assessment | C4.1 | 0.760 | 0.031 |
| AI-supported assessment | C4.2 | 0.820 | 0.027 |
| AI-supported assessment | C4.3 | 0.800 | 0.028 |
| AI-supported assessment | C4.4 | 0.750 | 0.032 |
| Critical judgement | C5.1 | 0.850 | 0.023 |
| Critical judgement | C5.2 | 0.820 | 0.026 |
| Critical judgement | C5.3 | 0.830 | 0.025 |
| Critical judgement | C5.4 | 0.870 | 0.021 |
| Educational ethics | C6.1 | 0.720 | 0.034 |
| Educational ethics | C6.2 | 0.780 | 0.030 |
| Educational ethics | C6.3 | 0.750 | 0.032 |

| Dimension | Item | Standardized loading | SE |
|---|---|---|---|
| Educational ethics | C6.4 | 0.800 | 0.028 |
| Disciplinary transfer | C7.1 | 0.810 | 0.027 |
| Disciplinary transfer | C7.2 | 0.840 | 0.024 |
| Disciplinary transfer | C7.3 | 0.790 | 0.029 |
| Disciplinary transfer | C7.4 | 0.820 | 0.026 |

*Note.* All standardized loadings were statistically significant at p ¡ .001. Loadings ranged from 0.720 to 0.870, indicating that the 28 items represented their intended latent factors well.

##### 1.4.1.1 Partial scalar invariance.

In the full scalar invariance model, the item intercepts for C2.1 and C7.1 showed notable cross-group differences. The partial scalar model therefore freed the intercept constraints for C2.1 ("I can write prompts that specify learning objectives, student level, output format, and constraints") and C7.1 ("I can adapt GenAI-supported tasks to the epistemic standards of my discipline"). The remaining 26 item intercepts were constrained equal across the Chinese and Kazakhstani samples.

#### 1.4.2 Supplementary Table S4b. Latent-factor correlations

Table S4b: Correlations among the seven latent readiness dimensions.

| Dimension | C1 | C2 | C3 | C4 | C5 | C6 | C7 |
|---|---|---|---|---|---|---|---|
| C1 Basic GenAI understanding | 1.000 | | | | | | |
| C2 Prompt design | 0.630 | 1.000 | | | | | |
| C3 AI-supported instructional design | 0.580 | 0.710 | 1.000 | | | | |
| C4 AI-supported assessment | 0.540 | 0.650 | 0.720 | 1.000 | | | |
| C5 Critical judgement | 0.660 | 0.690 | 0.660 | 0.640 | 1.000 | | |
| C6 Educational ethics | 0.490 | 0.520 | 0.550 | 0.610 | 0.680 | 1.000 | |
| C7 Disciplinary transfer | 0.560 | 0.670 | 0.700 | 0.660 | 0.710 | 0.600 | 1.000 |

*Note.* All latent-factor correlations were positive and statistically significant at p ¡ .001. Correlations ranged from 0.490 to 0.720 and did not exceed 0.850, supporting related but distinguishable readiness dimensions.

##### 1.4.2.1 Estimator, software, and missing-data treatment.

All statistical analyses were conducted in Python 3.11.9. Data cleaning and numerical computation used pandas 2.2.2 and NumPy 1.26.4. Descriptive statistics, group comparisons, regression models, and mixed-effects models used SciPy 1.13.1, pingouin 0.5.4, and statsmodels 0.14.2. CFA and measurement-invariance analyses used semopy 2.3.11. CFA used maximum-likelihood Wishart estimation with robust standard errors for parameter inference. Five-point Likert items were treated as approximately continuous. Measurement invariance was tested sequentially through configural, metric, scalar, and partial scalar models. Cronbach's alpha was calculated from the item covariance matrix, and McDonald's omega was calculated

from standardized loadings and residual variances. Because the final analysis sample had no missing values on the 28 readiness items, complete-case analysis was used and no imputation was performed.

## 1.5 Supplementary Table S5. Structured GenAI prompt-task training protocol

**Table S5.** Replicable protocol for structured GenAI prompt-task training.

| Session | Theme and delivery | Task | Required output |
|---|---|---|---|
| 1 | Course-objective prompt formulation; 90-minute onsite workshop. | Rewrite a course objective into a GenAI prompt specifying objective, student level, prior knowledge, duration, language, and output format. | Prompt draft; DeepSeek-R1 response; reflection on specificity. |
| 2 | AI-supported instructional design; 90-minute onsite workshop. | Generate a classroom activity for a real course, check disciplinary accuracy and learner fit, and revise the activity. | Revised activity sheet; faculty verification notes; reflection form. |
| 3 | AI-supported assessment; 90-minute onsite workshop. | Generate or revise a rubric, add process evidence, and specify student GenAI disclosure requirements. | Revised rubric; disclosure rule; evidence requirement; revision record. |
| 4 | Risk control and course policy; 90-minute onsite workshop. | Identify hallucination, bias, privacy, and overreliance risks in generated content and draft course-level AI-use rules. | AI-use rule; risk-control checklist; final reflection. |

*Note.* The four sessions were delivered once per week for four consecutive weeks, for a total of 360 minutes. Training was conducted as synchronous onsite workshops in computer classrooms or digital-teaching spaces at the five participating universities. Both the structured and conventional groups received four sessions and were kept comparable in contact time, learning environment, facilitator support, and access to basic training materials. The structured group used the prompt formulation–model-output review–course-material revision–written reflection cycle. The conventional group received GenAI function introduction, teaching-use cases, academic-integrity and privacy discussion, and general teaching-application advice, but did not complete the structured prompt-task cycle, output-verification record, rubric revision, or course-level AI-use rule drafting. The internal facilitator manual contained institution-specific official training arrangements; the replicable training sequence and standardized prompt wording are documented in Supplementary Tables S5 and S6.

#### *1.5.0.1 Training implementation conditions.*

Training tasks were completed through the official DeepSeek web interface using DeepSeek-R1, displayed in the web interface as "DeepThink (R1)." The concentrated access period was October 20–November 14, 2025. The study did not use the API, custom system prompts, or externally adjustable sampling parameters. The web-search function was kept off, and each training task used a new independent conversation to reduce carryover from previous interactions. Because the web interface did not expose an internal checkpoint or build number, model-version records included the interface display name, access date, access mode, and task timestamp.

Each participating university had one local facilitator, and two research-team members coordinated materials and implementation across sites. Local facilitators held at least a master's degree, had at least three years of higher education teaching, faculty development, or educational-technology training experience, and had prior experience with GenAI-supported teaching applications. Before formal training, facilitators attended a 120-minute protocol briefing and a 60-minute demonstration-task calibration. All sites used the same *GenAI Pedagogical Readiness Prompt-Task Training Manual, version 1.0* (manual ID: GPR-PTT-v1.0), task sheets, prompt templates, performance checklist, and written reflection form.

Facilitators explained task requirements, demonstrated basic operation, handled technical issues, reminded participants to remove sensitive information, and checked whether task materials were complete; they did not write or revise prompts on behalf of participants.

Participants could write prompts in Chinese, Kazakh, Russian, or English according to their course context. The research records retained original prompt and model-output language. English versions used for cross-national coding and manuscript reporting were translated and checked by bilingual researchers. Each task record included a de-identified participant ID, country and university code, session, task number, access date and time, full prompt, DeepSeek-R1 output, faculty verification record, revised teaching material, and written reflection. Stored data did not include faculty names, student names, student IDs, contact details, identifiable course names, or unnecessary personal or institutional identifiers.

## 1.6 Supplementary Table S6. Structured prompt templates used in the intervention

Table S6: Standardized structured GenAI prompt-task templates.

| Training task | Prompt template |
|---|---|
| Course-objective prompt | You are helping a university instructor redesign one course task. Course objective: [insert objective]. Student level and prior knowledge: [insert level and difficulties]. Course context: [insert discipline and class format]. Please propose a GenAI-supported learning task that aligns with the objective. Specify task steps, time allocation, expected student output, required resources, and limits on GenAI use. Do not assume student background beyond what is stated. |
| Instructional activity design | Design a [duration]-minute classroom activity for [student level] in [discipline/course topic]. The activity must address [learning objective], include [materials/constraints], and support students who may struggle with [learner difficulty]. Provide: (1) activity sequence, (2) teacher instructions, (3) student instructions, (4) expected output, and (5) verification points the teacher should check before classroom use. |
| Assessment and rubric revision | Revise the following assignment/rubric: [insert task or rubric]. Align it with [learning outcomes]. Add process evidence showing student reasoning, criteria for acceptable GenAI assistance, disclosure requirements, and prohibited uses. Return a rubric with criteria, performance levels, evidence requirements, and brief student-facing wording. |
| Faculty verification prompt | Review the generated teaching material below for factual errors, fabricated or unsupported claims, bias, privacy risk, disciplinary mismatch, and appropriateness for [student level]. Mark each concern, explain why it matters pedagogically, and suggest revisions that the instructor should verify before using the material. Material: [insert generated output]. |
| Student disclosure and AI-use rule | Draft a course-level student-facing rule for GenAI use in [course/discipline]. Separate uses into permitted, permitted with disclosure, and prohibited categories. Include examples, required disclosure wording, privacy reminders, and consequences for misuse. Keep the rule clear enough to include in a syllabus or assignment sheet. |

| Training task | Prompt template |
|---|---|
| Risk-control checklist | Create a checklist that a faculty member can use before adopting GenAI-generated content in a course. The checklist must cover hallucination checking, source verification, bias and fairness, student appropriateness, privacy/data-entry boundaries, assessment alignment, student disclosure, and disciplinary standards. Provide concise yes/no items and a final decision rule. |

*Note.* These templates provide the standardized prompt wording used to support replication of the structured GenAI prompt-task training. Faculty inserted their own course objectives, student level, disciplinary topic, constraints, generated material, or assessment task into the bracketed fields.

## 1.7 Supplementary Table S7. Prompt coding manual summary

Table S7: Coding manual for prompt strategy types and pedagogical themes.

| Code | Definition | Inclusion rule | Exclusion rule |
|---|---|---|---|
| Technical strategy | Prompt requests a concrete output format, such as a plan, steps, examples, or feedback. | Clear request for production, formatting, or procedural output. | No pedagogical purpose or course context. |
| Practical strategy | Prompt focuses on implementation, rubrics, rules, constraints, or course materials. | Includes concrete classroom, assessment, or policy use. | Pure exploration without implementation requirement. |
| Exploratory strategy | Prompt asks for alternatives, possibilities, comparisons, or brainstorming. | Requests multiple options or conceptual expansion. | Single fixed output with no alternatives. |
| Narrative strategy | Prompt asks for a classroom scenario, case, dialogue, or situated example. | Uses story-like or contextual framing. | Simple example list without situated scenario. |
| Structured strategy | Prompt requires a fixed template or multi-part output including checks or constraints. | Uses an explicit format such as objective, task, check, output, risk. | Loose prompt with no required structure. |
| Learning objectives | Prompt specifies learning outcomes, goals, or competencies. | Objective is explicit or requested. | General topic only. |
| Instructional activities | Prompt designs learning activities, classroom tasks, or teaching steps. | Activity sequence or teaching process is requested. | Assessment-only prompt. |
| Assessment design | Prompt concerns rubrics, criteria, feedback, grading, or evidence of learning. | Assessment criteria or feedback rules are present. | Generic learning activity without assessment. |

| Code | Definition | Inclusion rule | Exclusion rule |
|---|---|---|---|
| Critical thinking / judgement | Prompt asks students or faculty to compare, verify, critique, or justify. | Includes evaluation or reasoning demand. | Simple content generation. |
| Practical constraints | Prompt specifies time, level, materials, class size, language, format, or institutional condition. | Concrete constraint appears in prompt. | Broad prompt with no implementation boundary. |
| Structured prompting | Prompt uses a template or named sections. | Required output structure is specified. | Ordinary paragraph prompt. |
| Pedagogical innovation | Prompt seeks new methods, alternatives, or redesign. | Requests novel or alternative teaching design. | Routine procedural request. |
| Contextual application | Prompt adapts a task to a specific course, learner group, discipline, or classroom situation. | Context is named or described. | Generic output with no course context. |
| Learner difficulties | Prompt mentions student misconceptions, prior knowledge, language difficulty, or support needs. | Learner challenge is explicit. | Generic student level only. |
| Academic integrity | Prompt concerns disclosure, permitted use, prohibited use, process evidence, or misuse. | AI-use rule or integrity issue is explicit. | General ethics without course rule. |
| Risk control | Prompt asks for hallucination checking, bias detection, privacy boundary, or overreliance control. | Risk or verification step is explicit. | General quality improvement only. |

*Note.* Coding unit: prompt or prompt-revision segment. Strategy type was single-coded; pedagogical themes allowed multiple codes. Two coders double-coded 96 prompts (30%). Disagreements were resolved through discussion and final confirmation against this manual.

## 1.8 Supplementary Table S8. University-level sensitivity checks

Table S8: Estimability of university-level sensitivity checks.

| Check | Estimability issue | Reporting decision |
|---|---|---|
| University fixed effects for the RQ2 country coefficient | Country membership is perfectly nested within university because the five universities belong to either China or Kazakhstan. University fixed effects therefore absorb the country indicator. | Not reported as an inferential country-effect estimate. |

| Check | Estimability issue | Reporting decision |
|---|---|---|
| University-cluster robust standard errors for RQ2 | Conventional cluster-robust inference is unstable with five clusters. | Not used as primary evidence. |
| Training-group comparisons with university indicators | Training groups were organized through institutional training cohorts, and site-by-condition cells were small. | Reported as a descriptive robustness consideration rather than a primary estimator. |
| Prompt-design mixed model with university-level adjustment | The primary longitudinal model used participant-level random intercepts; adding university terms with five sites provides limited additional inferential leverage. | Summarized as an estimability note rather than a confirmatory cluster model. |
